# Ultra-Precision Manufacturing Technology for Difficult-to-Machine Materials

**DOI:** 10.3390/ma16124322

**Published:** 2023-06-11

**Authors:** Qi Liu, Mingjun Chen, Jian Cheng, Xichun Luo

**Affiliations:** 1 School of Mechatronics Engineering, Harbin Institute of Technology, Harbin 150001, China; q.liu@strath.ac.uk (Q.L.); cheng.826@hit.edu.cn (J.C.); 2 Centre for Precision Manufacturing, Department of Design Manufacturing & Engineering Management (DMEM), University of Strathclyde, Glasgow G1 1XJ, UK; xichun.luo@strath.ac.uk

Ultra-precision manufacturing requires superior components with an impeccable surface finish and accuracy. However, the need for ultra-precision equipment accuracy and problems in machining difficult-to-cut materials pose challenges. Specialized machine tools and understanding the process are vital to overcoming these challenges and improving the surface integrity and application performance.

This Special Issue comprises sixteen articles, including four studies on ultra-precision machining technology, four on tool design, four on non-traditional machining technology, and four on additive manufacturing.

In terms of ultra-precision machining technology, Yuanhang Liu et al. [1] developed an optimized grinding tool configuration and a comprehensive simulation model for wafer backside grinding, leading to effective total thickness variation control strategies. Marvin Groeb et al. [2] investigated the ductile regime face milling of sintered silicon carbide, revealing the influence of chip thickness and cutting speed on surface quality. Hongqing Lei et al. [3] developed a constitutive model considering KDP crystal anisotropy and utilized a 3D finite element model to analyze the impact of pre-existing micro defects during ball-end milling repair. Qi Liu et al. [4] performed fractal analysis on soft-brittle KDP crystal surfaces machined via micro ball end milling and identified fractal dimension as a means to distinguish between ductile and brittle material removal processes.

In terms of ultra-precision machine tool design, Zheng Qiao et al. [5] proposed an in situ measurement technique to solve the roundness error of a precision mandrel. By implementing the slow tool servo cutting technique, these errors were compensated and reduced to below 0.1 μm. Laiyun Song et al. [6] introduced a spiral-grooved structure to enhance the load capacity and stiffness of hybrid air journal bearings via frequency domain analysis. The study emphasized the significant influence of the spiral-groove length on spindle system stability. Additionally, Laiyun Song et al. [7] analyzed the nonlinear dynamic characteristics of the hybrid air bearing-rotor system at ultra-high speeds and theoretically investigated the rotor trajectory and non-linear behavior. Han Wang et al. [8] developed compliance equations for spatial elliptic-arc-beam spherical flexure hinges, providing a foundation for designing and modeling 3D elliptical vibration-assisted cutting mechanisms based on these hinges.

In terms of non-traditional precision machining technology, Boris Rajčić et al. [9] performed a picosecond laser treatment on nickel-based superalloy Nimonic 263 in different atmospheric conditions to enhance its service performance. The laser parameters and environmental conditions were optimized experimentally based on Taguchi’s robust parameter design. Jainlei Cui et al. [10] proposed a two-step laser machining process for PCD skiving cutters. A high-quality PCD skiving cutter was obtained with an Rt of 5.6 µm and no phase transition damage. Rakesh Chaudhari et al. [11] experimentally investigated the effect of alumina (Al2O3) nano-powder on the electrical discharge machining (EDM) process of a Nitinol shape memory alloy (SMA), and the related parameters were optimized via ANOVA analysis. Lida Heng et al. [12] summarized the recent advances in non-conventional finishing processes for difficult-to-machine ceramics.

In terms of precision additive manufacturing, Kaicheng Yu et al. [13] proposed a method to optimize the process parameters for improving the diameter accuracy of filaments extruded via 3D printing. Rongkai Tan et al. [14] adopted UEVC to cut SLM AlSi10Mg alloy post-process. The surface integrity and tool wear can be greatly improved. David Sommer et al. [15] investigated milling tool wear characteristics in a hybrid additive manufacturing process that comprises laser powder bed fusion and in situ high-speed milling, giving a potential solution for AM post-processing. Jian Cheng et al. [16] reviewed laser metal deposition for cladding from the defect formation mechanism to defect suppression methods. The performance improvements of laser cladding layers were also summarized.

Via this Special Issue, we hope that more scholars learn about the current research progress on the ultra-precision machining of difficult-to-machine materials and will be attracted to participate in it.
